# Aged related human skin microbiome and mycobiome in Korean women

**DOI:** 10.1038/s41598-022-06189-5

**Published:** 2022-02-11

**Authors:** Hye-Jin Kim, Han Na Oh, Taehun Park, Hanbyul Kim, Hyun Gee Lee, Susun An, Woo Jun Sul

**Affiliations:** 1 Department of Systems Biotechnology, Chung-Ang University, Anseong, South Korea; 2 Safety Research Team, Amorepacific R&D Center, Yongin, South Korea

**Keywords:** Microbial ecology, Microbial communities, Environmental microbiology

## Abstract

We examined differences in the skin microbiome of two separate age groups to find key microbial and skin physiological indicators associated with aging. We recruited healthy Korean women 19–28 years old (Y-group) and 60–63 years old (O-group) and evaluated their cheek and forehead skin microbiome, including bacteria and fungi. The microbiome was significantly different by age group, with bacterial and fungal communities displaying higher alpha-diversity in the O-group than in the Y-group. We identified amplicon sequence variants affiliated with *Cutibacterium* and *Lactobacillus* and fungi *Malassezia restricta* as microbial biomarkers showing significant differences between the Y and O-group. There are more microbial communities and metabolic processes related to skin health in the Y-group than in the O-group, and there are more microbial interactions to increase the stability of the network structure of the skin. Skin physical metadata, including transepidermal water loss and sebum content, differed by two age groups. The crucial skin microbes, skin physical parameters, and microbial network found through this research will be useful key indicators in associating skin aging and skin microbiome research.

## Introduction

The human skin microbiome, a complex ecosystem composed of bacteria, archaea, fungi, and viruses, is under the influence and affects host skin conditions^1,2^. Previous research revealed that specific microbes, such as *Cutibacterium acne* and *Staphylococcus aureus*, and host residences^3,4^, as well as physiological characteristics and immunity, may be associated with host skin health and disease status, such as acne, atopic dermatitis, and rosacea^5–7^. One of the most well-studied skin microbiome-related issues is skin aging, though it is not an official disease^8^. Skin aging is characterized by wrinkles, laxity, loss of elasticity, and the appearance of rough texture. These aging-related changes in the structure of the skin, as well as the accumulation of environmental influences during a person’s lifetime, increase the incidence of skin diseases^9,10^. The process of skin aging, accompanied by structural and functional changes to extracellular components and modification in skin cells, also leads to skin microbiome alteration. Several comparisons of skin microbiomes of younger (aged 20–30) and older women (aged 50–60) in Western Europe and Asia^11–14^ suggested a correlation between skin aging and skin microbiomes. In our previous study on Chinese women^14^, we described differences in the skin microbiome and inferred functional pathways by age. Nonetheless, the skin microbiome is often more variable due to ethnicity, gender, and residence location than to age.

Here, we conduct a clinical study, measuring the cheek and forehead microbiome and mycobiome. Since the cheeks and foreheads have different pH, temperature, and sebum content even for the same person^2^, microbiome and mycobiome analysis of two regions were performed, respectively. We focus on bacterial and fungal community structure and characteristics of healthy young and old Korean women. We identify the vital age-related bacteria and fungi found using amplicon sequence variants (ASVs) and reveal the functional properties of the skin microbiome by age. In addition, we review several similar studies to draw conclusions and suggest important indicators of the age-related skin microbiome.

## Results

### Study subjects and measurement of skin physiological parameters

We analyzed skin microbiome and mycobiome from cheeks and foreheads of healthy younger (19–28 years old, Y-group) and older (60–63 years old, O-group) Korean women who were free from cutaneous disorders (Table 1 and Supplementary Table S1). All 61 subjects had been living in Seoul, Korea, for more than 3 years with normal skin conditions. We preferentially selected those who had sebum secretion greater than 30 arbitrary units and moisture greater than 50 arbitrary units in both groups. Among the measurements of moisture content, pH, sebum content, and transepidermal water loss (TEWL), only sebum and TEWL decreased significantly in the O-group compared to the Y-group in the cheeks (*P* = 2.25e−06, Wilcoxon rank-sum test; *P* = 0.019, Welch two-sample *t* test) and forehead (*P* = 1.33e−06, Wilcoxon rank-sum test; *P* = 0.003, Welch two-sample *t* test). Whereas no significant differences were found in the average values for moisture (cheeks: Y-group, 59.9; O-group, 56.6; forehead: Y-group, 61.1; O-group, 58.7) and pH (cheeks: Y-group, 6.0; O-group, 5.8; forehead: Y-group, 6.0; O-group, 5.6) between the two age groups.

**Table 1:** Characteristics of subjects for aged related skin microbiome and mycobiome study.

Baseline characteristics	Healthy women	
Total number of subjects	61	
Age-group	Y-group (n = 29)	O-group (n = 32)
Age, mean (SD)	22.07 (2.31)	61.06 (0.98)
Sites of skin sample collected	Cheek (n = 15)	Cheek (n = 16)
	Forehead (n = 14)	Forehead (n = 16)

Physiological values	Mean (SD)	Mean (SD)
**Moisture**		
Cheek	59.42 (8.03)	58.03 (6.85)
Forehead	61.74 (6.11)	58.14 (7.06)
**pH**		
Cheek	6.00 (0.32)	5.82 (0.50)
Forehead	5.95 (0.56)	5.61 (0.51)
**Sebum**		
Cheek	40.17 (15.49)	4.13 (3.85)
Forehead	53.57 (20.38)	17.22 (11.49)
**TEWL**		
Cheek	21.99 (5.18)	18.19 (2.57)
Forehead	20.26 (3.49)	17.49 (3.59)

### Comparisons in cheek and forehead microbiome and mycobiome between the two age groups

We analyzed bacterial communities from 27 Y-group samples (cheeks, n = 13; forehead, n = 14) and 24 O-group samples (cheeks, n = 12; forehead, n = 12) and fungal communities from 28 Y-group samples (cheeks, n = 15; forehead, n = 13) and 32 O-group samples (cheeks, n = 16; forehead, n = 16), except for samples that were eliminated from the Illumina Mi-Seq sequencing due to low sequence reads (bacteria, < 10,000; fungi, < 1000 reads) (Supplementary Table S1). An average of 49,062 (Y-group) and 55,946 (O-group) merged sequences were obtained, and 5693 ASVs were generated and used for bacterial taxonomic assignments based on the SILVA database. An average of 12,478 (Y-group) and 11,055 (O-group) merged sequences were obtained, and 675 ASVs were generated and used for fungal taxonomic classification based on the UNITE database.

**Figure 1:**
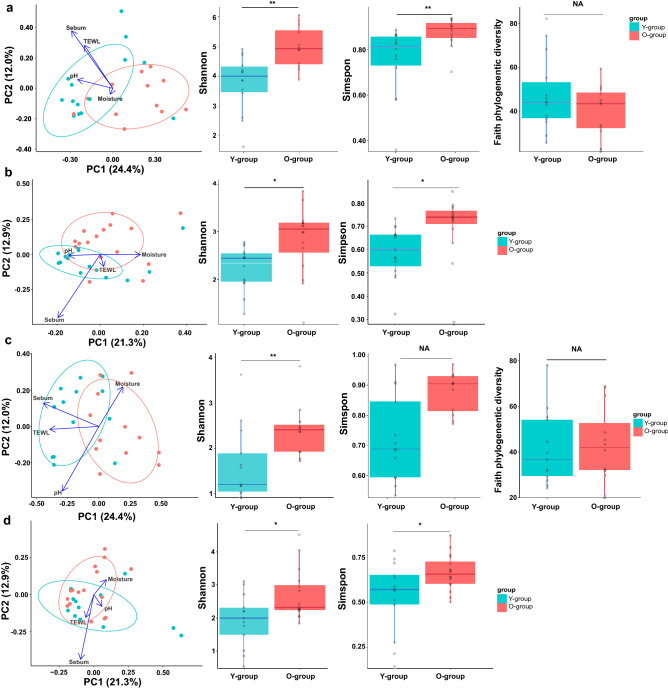
Characterization of skin microbiome and mycobiome in cheeks and forehead of Y-group and O-group. Principal coordinates analysis was performed based on Bray–Curtis distance between the Y-group and O-group in cheeks and forehead microbiome and mycobiome. (**a**,**b**) In the cheeks, the bacterial (**a**) and fungal (**b**) communities were significantly different by age (Y-group, *P* = 0.011 and O-group, *P* = 0.001; PERMANOVA). (**c**,**d**) In the forehead, the bacterial (**c**) communities show significant differences (*P* = 0.001, PERMANOVA), but the fungal (**d**) communities did not. Alpha diversity of bacterial and fungal communities on (**a**,**b**) cheeks and (**c**,**d**) forehead of the Y-group and O-group (***P < 0.001, **P < 0.01, *P < 0.05, using Wilcoxon rank-sum test and Welch two-sample *t* test).

Principal coordinates analysis with Bray–Curtis distances showed a clear separation in bacterial communities between the Y-group and O-group of cheek and forehead skin microbiome (Fig. 1) (*P* = 0.011, *P* = 0.001, respectively; PERMENOVA). The fungal communities showed significant differences between the Y-group and O-group only in the cheeks (*P* = 0.001; PERMANOVA). The alpha-diversity of bacterial and fungal communities was significantly higher in the O-group than the Y-group on both the forehead and cheek (Fig. 1). Simpson’s diversity analysis, which considers both richness and evenness, was significantly higher in the O-group cheek community, indicating that the skin microbiome and mycobiome of older women consisted of certain dominant microbes.

### Taxonomic overview and signatures of aged related microbial communities in cheek and forehead

We identified 36 bacterial and 6 fungal phyla from 61 skin samples. The most abundant bacterial phyla, Actinobacteria, Proteobacteria, Firmicutes, and Bacteroidetes, were present in the cheeks and foreheads of both the Y-group and O-group (Supplementary Fig. S1e,f).

**Figure 2:**
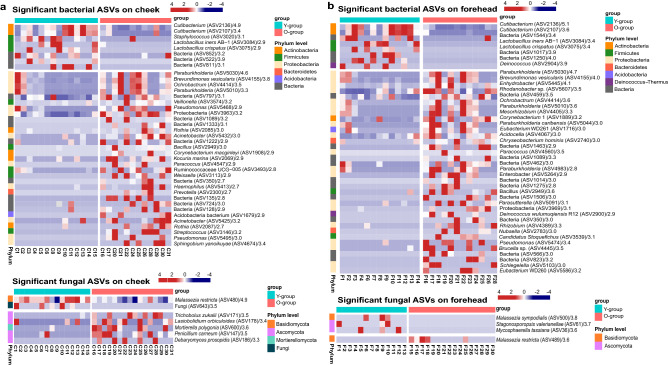
Heat map for the bacterial and fungal ASVs on the (**a**) cheeks and (**b**) foreheads of Korean women that shows a significant difference between the two age groups from LEfSe analysis (LDA score > 2.5 for bacteria, LDA score > 3.3 for fungi on the cheeks and LDA score > 2.7 for bacteria, LDA score > 3.5 for fungi on the foreheads).

We used the Linear discriminant analysis Effect Size (LEfSe) method to investigate the taxonomic biomarkers that contribute to age-related differences in the skin microbiome and mycobiome at the ASV level. In the cheek microbiome comparison, 39 bacterial ASVs were identified as preferentially abundant in one or the other age group: 8 ASVs in the Y-group and 31 ASVs in the O-group (Fig. 2a). *Cutibacterium* sp. (ASV2136 and ASV2107)*, Staphylococcus* sp. (ASV3020), *Lactobacillus iners* (ASV3084), and *Lactobacillus crispatus* (ASV3075) were predominant in the Y-group. The 31 ASVs found in the O-group belonged to more diverse phyla (Actinobacteria, Firmicutes, Proteobacteria, Bacteroidetes, and Acidobacteria) than those in the Y-group. The fungus *Malassezia restircta* (ASV480) was more abundant on cheeks in the Y-group, whereas *Trichobolus zukalii* (ASV171)*, Lasioboolidium orbiculoides* (ASV178)*, Mortierella polygonia* (ASV600), *Penicillium carneum* (ASV147), and *Debaryomyces prosopidis* (ASV186) were abundant on cheeks in the O-group.

In the forehead microbiome comparison, 44 bacterial ASVs were identified as preferentially abundant in one or the other age group: 8 ASVs in the Y-group and 36 ASVs in the O-group (Fig. 2b). *Cutibacterium* sp. (ASV2136 and ASV2107)*, L. iners* (ASV3084)*, L. crispatus* (ASV3075), and *Deinococcus* sp. (ASV2904) were found in the Y-group. As was true for the cheek microbiome comparison, the 36 bacterial ASVs identified in the O-group forehead microbiome belonged to more diverse phyla than those in the Y-group. Four fungal ASVs were identified in forehead samples: *Malassezia sympodialis* (ASV500), *Stagonosporopsis valerianellae* (ASV61), and *Mycosphaerella tassiana* (ASV36) were abundant in the Y-group, and *M. restricta* (ASV489) was abundant in the O-group (Fig. 2b). These results showed the dominant microbes were different according to whether cheek or forehead and whether Y-group or O-group.

### Identification of major physiological factors and associated microbes

**Figure 3:**
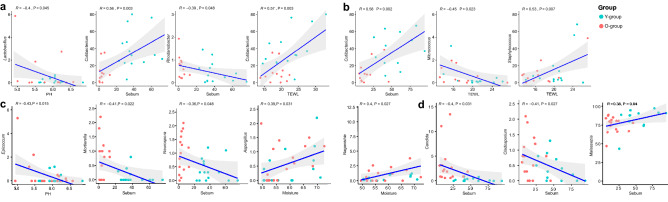
Regression plot for skin metadata and the bacterial and fungal genera with a significant correlation among the top 15 genera in (**a**,**c**) cheeks and (**b**,**d**) forehead. For measuring correlation coefficient and *P *value, Pearson correlation analysis was performed in R. The x-axis represents the value of metadata, and the y-axis corresponds to the relative abundance of the genus.

We performed a regression analysis using ggpubr package in R to determine the relationship between the skin microbes and skin parameters, with significant differences between the Y-group and O-group (Fig. 3). Regression analysis of the top 15 bacterial and fungal genera and skin parameters revealed that *Cutibacterium* in the cheek and forehead increased significantly with increasing sebum. In addition, *Cutibacterium* in the cheek showed a significant increase with increasing TEWL. In the forehead, it was confirmed that *Staphylococcus*, a dominant skin bacterium, increased significantly with increasing TEWL (*P* = 0.007; *t* test). In the case of fungi, *Mortierella* and *Neurospora* were found to correlate with a sebum reduction on the cheek (*P* = 0.022 and *P* = 0.048, respectively; *t* test) significantly. In the forehead, *Candida* and *Cladosporium* decreased with increasing sebum (*P* = 0.031, and *P* = 0.027, respectively). *Malassezia*, the dominant fungus in the skin microbiome, was found to have a significant correlation with an increase in sebum on the forehead (*P* = 0.04; *t* test). These results inferred that skin physiology influences the skin microbiome and increases or decreases the abundance of specific skin microbes.

### Functional profiles of cheek and forehead microbiomes

**Figure 4:**
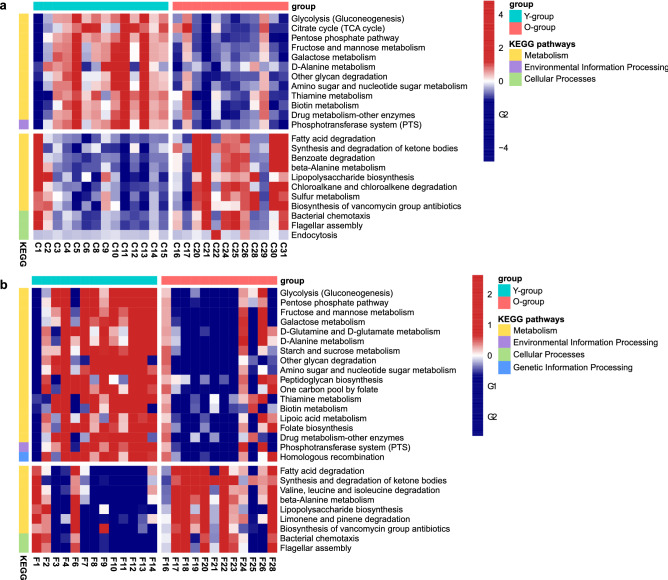
Heat map for significantly different predicted functional pathways on (**a**) cheeks and (**b**) foreheads of Korean women by age based on LEfSe analysis (LDA score > 3.0).

We performed Phylogenetic Investigation of Communities by Reconstruction of Unobserved States 2 (PICRUSt2) analysis to identify the potential functional differences in the microbial communities of the two age groups. From the cheek and forehead, respectively, 23 and 27 differentially abundant predictive metagenomic pathways, involved in the Kyoto Encyclopedia of Genes and Genomes (KEGG) categories of metabolism, environmental information processing, cellular processes, and genetic information processing, were identified (*α* = 0.05, LDA score > 3. 0) (Fig. 4). Pathways belonging to the metabolism category were dominant in each age group. In the cheek of the Y-group, pathways involved in energy metabolism by bacteria, such as glycolysis/gluconeogenesis, citrate cycle, pentose phosphate pathway, fructose and mannose metabolism, galactose metabolism, d-alanine metabolism, and thiamine metabolism, were predominant, whereas in the cheek of the O-group, degradation-related pathways, such as fatty acid degradation, synthesis and degradation of ketone bodies, benzoate degradation, and chloroalkane and chloroalkene degradation, were predominant. In the forehead of the Y-group, glycolysis/gluconeogenesis, pentose phosphate pathway, fructose and mannose metabolism, galactose metabolism, d-glutamine and d-glutamate metabolism, d-alanine metabolism, and thiamine metabolism pathway were significantly more abundant, whereas in the forehead of the O-group, fatty acid degradation, synthesis and degradation of ketone bodies, valine/leucine and isoleucine degradation, and limonene/pinene degradation pathway were significantly more abundant.

The metabolism pathway for biotin, a water-soluble vitamin that is effective for skin health and essential for keratin production^15^, was more prevalent in the cheek and forehead of the Y-group. Interestingly, the metabolism pathway for lipoic acid, which is known to possess beneficial effects against skin aging and is used widely in cosmetic and dermatological products^16,17^, was significantly higher in the foreheads of the Y-group. We tracked the specific ASVs possessing these pathways, in both biotin metabolism and lipoic acid metabolism, *Cutibacterium* sp. (ASV2136 and ASV2130) and *Staphylococcus* sp. (ASV3008) were predicted to have the top three relative abundances in KOs. The relative abundances in biotin metabolism and lipoic acid metabolism of *Cutibacterium* sp. (ASV2136) were 24.9% and 26.1%, respectively. The relative abundances for each pathway for *Staphylococcus* sp. (ASV3008) were 10.2% and 18.7%, and for *Cutibacterium* sp. (ASV2130), they were 9.3% and 10.0%, respectively. We confirmed these two pathways in the genome of skin bacteria, *C. acnes* (Supplementary Fig. S2). These additional analyses support the reliability of the function in the skin environment of *Cutibacterium.* Interestingly, from the LEfSe result, *Cutibacterium* sp. (ASV2136) had a significantly higher abundance in the cheek and forehead microbiome of the Y-group. The pathway of biosynthesis of lipopolysaccharide, also known as bacterial endotoxins, showed higher abundance in the cheek and forehead microbiome of the O-group. The ASVs that contribute to inferring the LPS biosynthesis pathway were identified as *Paraburkholderia* sp. (ASV5030) and *B. vesicularis* (ASV4155). Also, pathways related to antibiotic biosynthesis (biosynthesis of vancomycin group antibiotics) and bacterial motility (bacterial chemotaxis and flagellar assembly; both belonging to the cellular processes category) were prominent in the cheek and forehead of the O-group. PICRUSt2 analysis implied that, regardless of skin site differences, the potential functions of the microbial community that compose the skin microbiome were similar according to age.

### Network analysis on cheek and forehead microbiome and mycobiome

**Figure 5:**
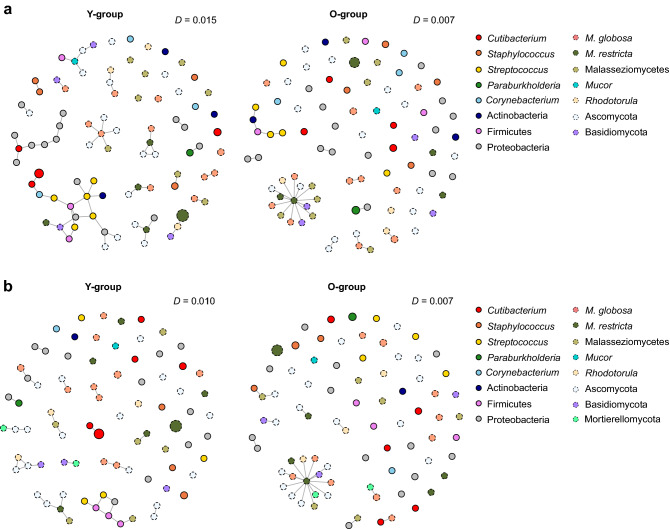
Network analysis of the ASVs on (**a**) cheeks and (**b**) forehead of Korean women. Each node represents the ASV and the size of the node is based on relative abundance of each ASV. Color markings indicate the major taxa except for unidentified bacteria or fungi. Shapes represent the level of kingdom, Bacteria (bold) and Fungi (dotted line). The ASVs were selected for bacterial ASVs found in more than half of all samples on the cheeks and forehead, respectively, and for the fungal ASVs with a relative abundance of more than 0.1% in each of the cheeks and forehead samples. The *D* value is network density calculated using the ratio of the number of edges.

We performed SParse InversE Covariance estimation for Ecological Association Inference (SPIEC-EASI) analysis to evaluate the overall network of the skin microbes. The results of network density (*D*) on 81 cheek and 87 forehead ASVs, calculated using the ratio of the number of edges, showed higher network density in the skin microbiome of the Y-group (*D* = 0.015 and *D* = 0.001, in cheek and forehead, respectively) than the O-group (*D* = 0.007 and *D* = 0.007, respectively) (Fig. 5). To examine network correlation between bacteria and fungi, network density for Bacteria–Fungi (*D*_BF_) was calculated by the actual number of edges and a potential number of edges in a correlation ([bacterial nodes × fungal nodes]/2). We confirmed higher network density in the cheek of the Y-group (*D*_BF_ = 0.008) than the O-group (*D*_BF_ = 0) and edges of the major bacterial and fungal taxa, such as *Staphylococcus* sp. (ASV3008)—*M. sympodialis* (ASV500) and *Roseomonas* sp. (ASV4088)—*M. restricta* (ASV482), were observed in the cheek of the Y-group. In the forehead, edges of *Methylobacterium* sp. (ASV4314)—*M. globosa* (ASV454), *Methylobacterium* sp. (ASV4314)—*Zygosaccharomyces rouxii* (ASV208), and *Venionella* sp. (ASV3575)—*M. sympodialis* (ASV500) were observed in the Y-group, and edges of *Cutibacterium* sp. (ASV2107)—*M. globosa* (ASV461), *Staphylococcus* sp. (ASV3024)—*M. arunalokei* (ASV446), and *Methylobacterium* (ASV4314)—*M. dermatis* (ASV448) were observed in the O-group (*D*_*BF*_ = 0.004). We found a network between bacteria and fungi with different kingdom levels in the skin microbiome, and especially, we confirmed that different genus or species level microbe was involved in the microbial network according to skin location and Y-, O-group.

## Discussion

Skin aging is not a formal skin disease; however, it is an unavoidable process for all humans. It has been a critical issue for people because it involves highly visible physiological changes^18^. The skin microbiome exhibits different characteristics according to various factors such as ethnicity, age, gender, and location of residence, but not enough research has been conducted to understand the relationship between skin aging and the microbiome. In this study, we examine the age-related characteristics of skin microbiomes and review several similar previous studies.

Bacterial and fungal communities of the Korean women in this study were distinguishable by age group. Other recently published studies have shown segregation between age groups; however, the microbial community features revealed in each study are inconsistent (Supplementary Table S2). Juge et al.^19^ compared the forehead microbiome of Eastern European females in two age groups (younger group, 21–31 years old; older group, 54–69 years old). They found the older individuals had higher alpha diversity, with higher relative abundances of *Corynebacterium* as well as the lower abundance of *Cutibacterium* compared to the younger individuals. Shibagaki et al*.*^12^ also reported that the alpha diversity and species richness of the cheek and forehead microbiome of Japanese women were significantly higher in the older group (60–76 years old) than in the younger group (21–37 years old). Despite the similar age ranges, our previous study comparing cheek microbiomes in Chinese women showed the opposite pattern, with skin microbiome in the younger group having higher diversity than that in the older group. Comparative research of skin microbiome in seven skin sites of Chinese adolescents (12–19 years old), younger adults (25–35 years old), and older adults (50–60 years old) showed that the younger adult's group maintained a greater overall diversity compared to adolescent and older adult groups^11^. In the current study, the alpha diversity of bacterial and fungal communities was significantly higher in the O-group. Studies of skin microbiome related to age show variable results on a case-by-case basis. This suggests that comparisons of taxonomy- or phylogeny-based diversity of bacterial communities were not sufficient for distinguishing younger and older skin microbiomes. Thus, additional analysis and strategies are needed to understand the structure and characteristics of the skin microbiome as it relates to age.

In the present study, from the comparison of significantly different ASVs, *Cutibacterium*, *Paraburkholderia,* and *Streptococcus* were predominant in the cheeks, and *Cutibacterium*, *Staphylococcus,* and *Paraburkholderia* were more abundant in the forehead of the O-group. Among fungal species, *Malassezia* and *Rhodotorula* dominated the cheeks and forehead of the O-group. Even though varieties of age-specific microbes were present on the forehead and cheeks, the inferred metagenomic pathways were similar in each age group. The fungi found in the LEfSe analysis were not as diverse as the bacteria, but the cheeks and forehead host different fungi, which implies that the presence and role of fungi and interaction between fungi and bacteria must be taken into account in the study of the skin microbiome.

As reported in a previous age-related skin microbiome study of Chinese women^14^, we found that sebum and TEWL were significantly different in the two age groups. The significant decrease of sebum and TEWL related to skin barrier function influenced by skin aging has been reported^18,20^. Despite differences in average skin physical parameters between Chinese and Korean women, the significant reduction of sebum and TEWL in the older individuals’ skin suggests that these two factors are important indicators in the relationship between the skin microbiome and aging.

In microbial network analysis, we found that density (*D*) and transitivity (*T*) (also known as a clustering coefficient), were higher in the younger group, suggesting that the skin microbiome had a complex inter-microbial network. In particular, the higher network density between bacteria and fungi (*D*_BF_) and the appearance of the edges of the major bacterial and fungal taxa in the Y-group implies that the microbial network of the younger skin microbiome is more complex and indicates the importance of the inter-domain microbial networks in the skin. Through network analysis of the skin microbial communities, it was confirmed that genera, which are known to play an important role in the skin microbiome and mycobiome, act as the main hub in the microbial network. In addition to our findings on the association between skin-care products and the microbiome, we found a connection between low-abundance bacteria and fungi, such as *M. sympodialis*, *Gemella,* and *Methylobacterium*, which are rarely mentioned in other skin microbiome research. According to Chng et al.^21^, *Methylobacterium* was found as one of the differentially abundant genera in control skin compared to skin affected by atopic dermatitis. Leung et al*.*^22^ discuss how, through network analysis, members of the microbial communities of the skin microbiome, as well as their co-abundance and co-exclusion relationships, can be observed. They also discuss how the potential importance of the low-abundance genera in the skin can be highlighted through network analysis^22^. The effect of these low-abundance but critical microbes in the skin ecosystem has yet to be examined, suggesting future studies of their potential roles in skin aging. When the skin microbiomes of age groups are assessed, archaea are more abundant in subjects over 60 years old and children under 12 years old than in middle-aged human subjects^23^. These insights suggest further study of factors in the skin ecosystem that have not been considered in skin microbiome research.

The information on age group-specific key microbes, skin parameters as major indicators, and microbial networks will provide building blocks in subsequent studies of skin aging and skin microbiome and mycobiome. Directly comparing age-specific bacterial and fungal communities in one case study and relatively small sample size may be insufficient for understanding the relationship between skin microbes and the causes or effects of skin aging. Thus, additional research into the role of both skin microbiome and mycobiome in skin aging is required.

## Methods

### Study design and sample collection

We recruited 32 female volunteers for the study, of which 16 were aged 20–29 years and 16 were aged 60–69. Through the survey, women of two age groups were grouped into Y- and O-groups. In order to select suitable subjects, we measured the sebum and moisture in advance and skin condition was checked by a dermatologist. All procedures were performed in a temperature- and humidity-controlled room (22 ± 2 °C and 50 ± 10% RH). On the day of the test, each subject visited without washing her face. Skin samples were collected from the subject's right cheek and forehead using sterile cotton-tipped swabs (COPAN ref. 165KS01), 0.15 M sodium chloride (NaCl), and 0.1% tween 20. When the microbial collection was completed, each subject washed her face and waited for more than 30 min in the temperature- and humidity-controlled room. Skin moisture content, sebum content, pH, and TEWL were measured on the subject's right cheek and forehead. Moisture content was measured three times using the Corneometer CM 825 (Courage + Khazaka Electronic GmbH, Cologne, Germany), and sebum content was measured twice with Sebumeter SM 810 (Courage + Khazaka Electronic GmbH). The skin surface pH was measured twice using a pH-meter (Courage + Khazaka Electronic GmbH), and TEWL was measured twice using a Vapometer (Delfin Technologies, Kuopio, Finland). We took photographs of all subjects in order to confirm their skin condition.

The skin microbiome sampling and skin measurement protocol presented by Amorepacific were approved by Institutional Ethics Review Board of CRO, Institut d'Expertise Clinique Korea, approval number IECK (1)-IRB- 18K041326). This study was conducted according to Good Clinical Practice (GCP) guidelines, International Conference on Harmonization (ICH) research guidelines, and in accordance with the principles of the Declaration of Helsinki and its later amendments. The part of participants recruiting, skin microbiome sampling, and skin measurement were performed by Amorepacific through contract research, and all of these clinical steps were conducted at the IECK. All participants provided their written informed consent, including for publication of results, after a full explanation of the protocol.

### Bacterial and fungal gDNA extraction

Bacterial and fungal gDNA extraction was carried out using the gram-positive bacterial cell lysate procedure of the PureLink^®^ Genomic DNA Mini Kit (Life Technologies, Carlsbad, CA, USA)^24^. In brief, a lysis buffer containing 20 mg/mL lysozyme was added to each swab sample, after which the tube was vortexed briefly to obtain the lysate. Proteinase K was added at a volume equivalent to one-tenth of the lysis buffer, followed by 445 µL of genomic lysis/binding buffer. Next, two stainless steel beads (5 mm, Qiagen, Hilden, Germany) were placed in each tube, and then bead-beating was performed for 1 min using a Bead Beater 16 device (Bio Spec Products Inc., Bartlesville, OK, USA). The tubes were then cooled on ice for 10 min and incubated at 55 °C for 30 min. Finally, after a washing process, the gDNA was extracted by elution with 30 µL of PureLink^®^ Genomic Elution Buffer and stored at − 20 °C until sequencing. The concentration and purity of the gDNA were measured using a NanoDrop 2000 spectrophotometer (Thermo Fisher Scientific Inc., Waltham, MA, USA).

### Polymerase chain reaction (PCR) and 16S rRNA gene sequencing

For each bacterial gDNA sample, the v4–v5 region of the 16S rRNA gene, which can cover most of the described human bacterial diversity and provide taxa information that are under-represented in skin microbiome surveys using other hypervariable regions, was amplified using the 518F-926R primer fused with a barcode (N701-N715/S502-S511)^24^. The forward primer included the Illumina sequencing primer (5′-TCG TCG GCA GCG TCA GAT GTG TAT AAG AGA CAG CCA GCA GCY GCG GTA AN-3′) and the reverse primer included the Illumina pre-adapter (5′-GTC TCG TGG GCT CGG AGA TGT GTA TAA GAG ACA GCC GTC AAT TCN TTT RAG T-3′). For each fungal gDNA sample, the fungal internal transcribed spacer 1 DNA region of 18SF–5.8S 1R was used with the barcode and Illumina sequencing primer. The amplification reaction comprised initial denaturation at 95 °C for 3 min, followed by 25 cycles of amplification (denaturation at 95 °C for 30 s, annealing at 55 °C for 30 s, and elongation at 72 °C for 30 s), and a final extension at 72 °C for 5 min. The amplified products were then purified using AMPure XP beads (Beckman Coulter, High Wycombe, UK). An index PCR was performed under the same conditions as the amplification procedure except that eight cycles of amplification were used. The obtained DNA was subjected to quality assessment using PicoGreen dye and a NanoDrop spectrophotometer. The final purified product was quantified by quantitative PCR (qPCR) according to the qPCR Quantification Protocol Guide of the KAPA Library Quantification kits for the Illumina sequencing platform. Additionally, the product was determined using the LabChip GX HT DNA High Sensitivity Kit (PerkinElmer, Waltham, MA, USA). The final samples were sequenced on the Illumina MiSeq™ platform (Illumina, San Diego, CA, USA) as paired-end (2 × 300 bp) reads.

### Bacterial community analysis

The MiSeq raw demultiplexed reads were imported into QIIME2-2019.4^25^ and the v4–v5 primer sequences were trimmed using the program Cutadapt^26^. The trimmed reads were merged, denoised at 252-bps position and 182-bps position for forward and reverse reads, respectively, and clustered by ASVs using the program DADA2^27^. A total of 46,822,275 merged sequences and 5693 ASVs were classified taxonomically at each level with the QIIME2 feature-classifier against the SILVA-132-99 database^28^. The ASVs classified as mitochondria, chloroplast, archaea, and unassigned were filtered, and a phylogenetic tree was constructed from the final trimmed sequences. All of the sample sequences were rarefied to the depth 10,000 for microbial diversity analysis. Shannon diversity, Faith phylogenetic diversity, and Simpson diversity indices were used to obtain the alpha diversity calculation. The beta diversity was estimated using Bray–Curtis distance from the rarefied ASVs table.

### Fungal community analysis

The obtained MiSeq sequences were imported into the QIIME2-2019.4 and the internal transcribed spacer 1 region sequences were trimmed using itsxpress trim-pair-output-unmerged^29^. The trimmed paired-end sequences were denoised, filtered, and merged by DADA2 with a quality score over 20. We obtained 1,364,490 merged sequences and 675 ASVs from this process. For taxonomic classification on the denoising ASVs, feature-classifier QIIME2 script was conducted against the UNITE database (version 8.3, 2021.10)^30^. The sequences rarefied to depth 1000 alpha diversity and beta diversity were calculated using the Shannon diversity and Simpson diversity indices, and Bray–Curtis distance, respectively.

### Functional profiling prediction by PICRUSt2

The prediction for functional profiles from 16S rRNA gene sequences data was conducted using PICRUSt2 (v.2.1.3-b) software^31^, which predicts pathway abundance based on metagenome profiles. From the representative ASVs sequences, predicted functional profiles were obtained using the PICRUSt2 script with default options (picrust2_pipeline.py) and the KEGG pathway abundances from the predicted KO abundances with the ‘no_group’ option (pathway_pipeline.py) were inferred. Afterward, we tracked the ASVs having the KOs using the script with the ‘strat_out’ option (metagenome_pipeline.py). To find the specific ASVs that quantitatively possessed these pathways, we tracked the KEGG Orthology (KO) that comprises each pathway on the KEGG website. Then we calculated the relative abundance of ASVs with the corresponding KOs. To support the PICRUSt2 results, we checked whether there are actually genes belonging to the pathway in the genome. For identifying the species of *Cutibacterium* sp. ASVs (ASV2136 and ASV2107), which led to significantly different results in the picrust2, the sequences of two ASVs blasted into the NCBI 16S-ITS database, and the strains with the top hit of bit-score were obtained. We found the lowest common taxon for this strain and confirmed that ASV2136 and ASV2107 were *C. acnes.* Then, we searched 283 *C. acnes* genomes in the PATRIC database to identify whether there are genes belonging to biotin metabolism and lipoic acid metabolism in the genomes of *C. acnes*^32^.

### Network analysis on bacterial and fungal microbiome

To infer the skin microbial networks from the cheeks and foreheads of two groups, the microbiome relative abundance data (bacterial ASV table and fungal ASV table) and SPIEC-EASI package were used^33^. The bacterial ASVs with a frequency of ≥ 50% were used and excluded those with an abundance of zeros in the samples of each group. The fungal ASVs with a relative abundance of more than 0.1% in samples of each group were selected. A new ASV table was created by selecting only common samples having both bacterial ASVs and fungal ASVs. Finally, 87 ASVs on cheeks and 81 ASVs on foreheads were used for the network analysis. We ran the sparse neighborhood algorithm and model selection using the Stability Approach to Regularization Selection (StARS) method^34^ with a variability minimum lambda (λ) threshold of 0.05%. All steps in the network analysis were processed using the R package SpiecEasi:0.1.2 and the igraph package.

### Statistical methods

To evaluate the significant difference between the bacterial communities and fungal communities of the two groups, PERMANOVA was used on the Bray–Curtis distance matrices in the QIIME script with 999 permutations. The envfit test was performed using the envfit function from vegan package in R for fitting skin metadata onto the beta diversity ordination from Bray–Curtis distance matrices. We used Pearson’s correlation and applied linear regression^35^ for analyzing the correlation between skin metadata (moisture, sebum, pH, and TEWL) and relative abundance of the top 15 genera. The correlation coefficients (r) and p values were calculated and visualized using ggscatter from the ggpubr package in R.

The Shapiro–Wilk test (shapiro.test) for normality and nonparametric Mann–Whitney test (wilcox.test) for estimating the significance of alpha diversity values were used in R. To obtain significantly different taxonomic and predicted functional features of cheek and forehead microbiomes between the Y- and O-group, the LEfSe algorithm was used^36^. For testing all features in different groups, an alpha value of 0.05 for the factorial Kruskal–Wallis test was used, and for the resulting subset features, LDA scores of 2.0 were applied.

## Supplementary Information

Supplementary Figure S1.

Supplementary Figure S2.

Supplementary Table S1.

Supplementary Table S2.

## Data Availability

Sequence data from this study have been archived at the NCBI Sequence Read Archive (SRA) under BioProjects: PRJNA613934 and PRJNA614620.

## Abbreviations

ASV: Amplicon sequence variant
KEGG: Kyoto Encyclopedia of Genes and Genomes
KO: KEGG Orthology
LEfSe: Linear discriminant analysis effect size
PICRUSt2: Phylogenetic Investigation of Communities by Reconstruction of Unobserved States 2
TEWL: Transepidermal water loss
